# Racioethnic Differences in Human Posterior Scleral and Optic Nerve Stump Deformation

**DOI:** 10.1167/iovs.17-22141

**Published:** 2017-08

**Authors:** Ehab A. Tamimi, Jeffrey D. Pyne, Dominic K. Muli, Katelyn F. Axman, Stephen J. Howerton, Matthew R. Davis, Christopher A. Girkin, Jonathan P. Vande Geest

**Affiliations:** 1 Department of Bioengineering, University of Pittsburgh, Pittsburgh, Pennsylvania, United States; 2 Department of Mechanical Engineering, University of California Berkeley, Berkeley, California, United States; 3 Department of Aerospace and Mechanical Engineering, University of Arizona, Tucson, Arizona, United States; 4 Department of Ophthalmology, University of Alabama Birmingham, Birmingham, Alabama, United States; 5 McGowan Institute for Regenerative Medicine, University of Pittsburgh, Pittsburgh, Pennsylvania, United States; 6 Department of Ophthalmology, University of Pittsburgh, Pittsburgh, Pennsylvania, United States; 7 Louis J. Fox Center for Vision Restoration, University of Pittsburgh, Pennsylvania, United States

**Keywords:** glaucoma, sclera, strain, race, ethnicity

## Abstract

**Purpose:**

The purpose of this study was to quantify the biomechanical response of human posterior ocular tissues from donors of various racioethnic groups to better understand how differences in these properties may play a role in the racioethnic health disparities known to exist in glaucoma.

**Methods:**

Sequential digital image correlation (S-DIC) was used to measure the pressure-induced surface deformations of 23 normal human posterior poles from three racioethnic groups: African descent (AD), European descent (ED), and Hispanic ethnicity (HIS). Regional in-plane principal strains were compared across three zones: the optic nerve stump (ONS), the peripapillary (PP) sclera, and non-PP sclera.

**Results:**

The PP scleral tensile strains were found to be lower for ED eyes compared with AD and HIS eyes at 15 mm Hg (*P* = 0.024 and 0.039, respectively). The mean compressive strains were significantly higher for AD eyes compared with ED eyes at 15 mm Hg (*P* = 0.018). We also found that the relationship between tensile strain and pressure was significant for those of ED and HIS eyes (*P* < 0.001 and *P* = 0.004, respectively), whereas it was not significant for those of AD (*P* = 0.392).

**Conclusions:**

Our results suggest that, assuming glaucomatous nerve loss is caused by mechanical strains in the vicinity of the optic nerve head, the mechanism of increased glaucoma prevalence may be different in those of AD versus HIS. Our ONS strain analysis also suggested that it may be important to account for ONS geometry and material properties in future scleral biomechanical analysis.

Glaucoma is a neuronal degenerative disease that damages retinal ganglion cells (RGC), leading to irreversible vision loss.^1^ In 2010, glaucoma affected 60.5 million people, a number that is projected to rise to approximately 80 million by 2020.^2^ Risk factors for glaucoma include family history of glaucoma, race, age, and increased intraocular pressure (IOP).^1,3,4^ These factors influence the biomechanical environment of the lamina cribrosa (LC), which is hypothesized to be the primary region of mechanical insult in glaucoma.^3,5–8^ Changes in tissue properties of the optic nerve head and surrounding sclera are believed to accompany the initiation and progression of glaucoma by either influencing LC deformations or by causing a reduction of ocular blood flow.^9–16^ Open angle glaucoma (OAG) has been shown to be more prevalent in African descent (AD) populations at all ages, but proportionally increases more rapidly in the European descent (ED) population over the same age compared with AD populations.^4^ Stiffness of peripapillary (PP) sclera surrounding the optic nerve has been reported to increase with age and in those of AD compared with those of ED.^17,18^ Significant microstructural differences in human posterior scleral tissue are also present between these two racioethnic groups, with those of AD having less equatorially aligned collagen fibers compared with those of ED.^17,19^ These microstructural and material property differences may be responsible for the varying rates of glaucoma occurrence observed between those of AD and ED. In addition, the prevalence of OAG for those of HIS was found to be higher compared with those of ED, but lower compared with those of AD.^20^ To the authors' knowledge, no experimental biomechanical characterization of Hispanic descent (HIS) posterior ocular tissues has been reported in the literature.

From these prior studies, it is important to determine whether there exist any fundamental biomechanical differences in nonglaucomatous eyes in posterior ocular tissues across races and posterior eye regions. Experimental determination of the biomechanical properties of the enucleated optic nerve stump (ONS) and sclera is challenging due to their nonlinear and anisotropic response, as well as their complex geometry. High-resolution deformation protocols of the posterior sclera that use pressure-inflation experiments have been reported, with three-dimensional (3D) digital image correlation (DIC),^21–23^ 3D ultrasound speckle tracking (UST),^24–28^ and electronic speckle pattern interferometry (ESPI),^29–33^ being among the most commonly used full-field optical techniques. ESPI offers very high scleral deformation sensitivity,^29,32^ whereas 3D DIC has achieved high-resolution measurement of in-plane scleral displacement.^21,23^ 3D UST has been used to acquire volumetric scans quantifying complex internal scleral strains.^27^ Despite their numerous utilization in scleral deformation studies, none of these 3D deformation measuring techniques have been used to quantify human ONS displacement, due to current measurement limitations and the aspherical geometry of posterior ocular tissues.^21^ This complex geometry demands proper contouring and matching of the local shape with the displacement measurement to enable correct strains map calculation.^21,34,35^ For ESPI, these geometry deviations lead to shadows from oblique illumination that results in irregular data analysis.^36^ In standard DIC techniques, such complex nonspherical geometries with sharp angles are not fully captured in the two viewing angles leading to improper correlation and reconstruction. Because these angles cannot be readily changed without influencing in-plane and out-of-plane resolution,^37^ data analysis from these methods is not accurate for these complex geometry regions.

Our research group recently developed an S-DIC method with advanced 3D capabilities to map posterior sclera and ONS deformations.^37^ In this method, we improved the *z* (in-depth) resolution without loss of *x-y* (in-plane) sensitivity in DIC measurements. Our approach used one high-resolution camera that recorded videos of a pressure inflation device as it moved through two orthogonal parallax axes using a standard 3D DIC approach to quantify the deformation of posterior sclera and ONS across carefully selected sequential movie frames (up to the frame rate of the camera). The capabilities of the S-DIC technique were assessed in a prior work by the measurements of shape and displacement on a rigid complex geometry (ONS surrogate) with a reconstruction accuracy of 0.17% and 8-μm uncertainty in the out-of-plane direction.^37^ There are few studies in the literature that have studied the biomechanical properties of the ONS.^13,15,38–41^ As for comparisons across racioethnic groups, only variation of laminar depth with age^42^ and LC displacement^43^ in normal eyes across AD and ED groups have recently been reported. The racioethnic variation in IOP-induced deformation of the human ON in nonglaucomatous tissue has not been reported in the literature, in particular for those of HIS. The purpose of this work was to use S-DIC to evaluate the strains of nonglaucomatous scleral shells across three racioethnic groups (AD, ED, and HIS), four regions (temporal [T], nasal [N], superior [S], and inferior [I]), and three zones (PP sclera, non-PP sclera, and ONS). This comparison was done by analyzing data from four IOP states: 5, 15, 30, and 45 mm Hg.

## Materials and Methods

### Posterior Scleral Shells and ONSs

Posterior scleral shells from human donors of AD (57 to 98 years old, *n* = 7 eyes), of ED (52 to 92 years old, *n* = 11 eyes), and HIS (56 to 95 years old, *n* = 5 eyes) were received from the Alabama Eye Bank in Birmingham, AL, USA; the Banner Sun Health Research Institute in Sun City, AZ, USA; the Donor Network of Arizona in Phoenix and Tucson, AZ, USA; the Illinois Eye Bank in Chicago and Bloomington, IL, USA; the Michigan Eye Bank in Ann Arbor, MI, USA; and the San Diego Eye Bank in San Diego, CA, USA. All eyes were classified as nonglaucomatous according to available medical paperwork, next-of-kin questionnaires, and/or later confirmed via optic nerve axon count. Age of all donors for this study was limited to those over 50 years of age. The eyes were kept on ice until they were shipped to our laboratory and were stored in physiologic saline at 4°C. For axon counting, the optic nerves corresponding to the scleral shells were cut and fixed immediately in Poly/LEM (Polyscience, Inc., Warrington, PA, USA) at their respective eye banks, at which point they were sent to our laboratory. Once received, the nerves were transferred to vials containing 2.5% glutaraldehyde, in which they stored for 24 hours, transferred to PBS, and stored at 4°C until they were sent out for processing. For processing, two to three 1-mm-thick slices were cut from the middle of the available axial length of each nerve. In a few cases, a clean slice could not be prepared, and these nerves were left intact. The slices were washed several times with PBS and fixed with 1% osmium tetroxide in PBS for 2 hours on ice. The samples were extensively rinsed with ddH_2_O, dehydrated through a grades series of ethanol concentrations, treated with propylene oxide, and infiltrated with Eponate 12 resin overnight (Ted Pella, Redding, CA, USA). The following day, the slices were placed in fresh resin, and polymerized at 60°C overnight. Semi-thin sections were cut from the blocks on an Ultracut E ultramicrotome (Reichert-Jung, Wein, Austria) and stained with 1% toluidine blue in 1% sodium borate. Unfortunately, some of the embedded slides lacked the necessary quality for axon counting and were not used. The Table reports the race, age, sex, and anatomical location of all donor eyes, including whether or not the optic nerve cross section was used for axon counting.

**Table:**
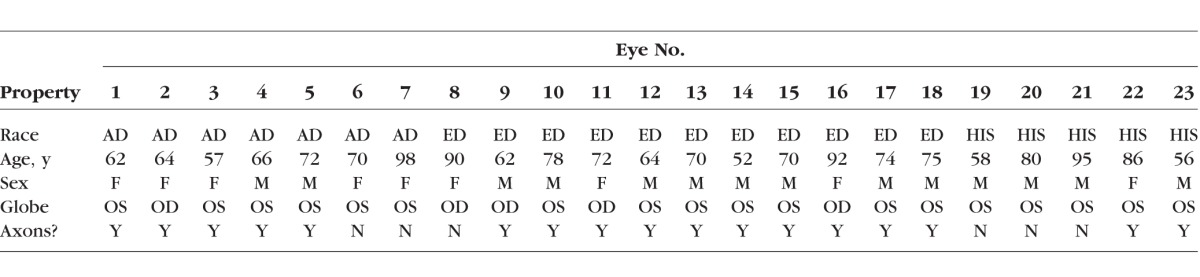
Donor Race, Age, Sex, Anatomical Location, and Axon Count Availability of All Donor Eyes Used in This Study

### Automated S-DIC

All scleral shells were tested using an S-DIC inflation procedure previously developed by our research group.^37^ As part of our research group's efforts to enhance our previously reported S-DIC imaging approach, significant improvements were made to the system to reduce hands-on imaging time, maintain consistent eye hydration, upgrade vibration control, and streamline postprocessing methods. Overall, the most significant improvement to the S-DIC system was the inclusion of automated motor rotational stages (CR1-Z7, PRM1Z8; Thorlabs, Newton, NJ, USA), controlled by LabView (National Instruments, Inc., Austin, TX, USA) that acted as the primary controller that managed the camera acquisition and rotational stage movements throughout the experiments. The inclusion of the automatic motors allowed for improved vibration control due to the fine incremental rotational motion resolution, as well as eliminated the need to open the humidity chamber doors for manual actuation thus improving humidity consistency. All other S-DIC experimental protocol steps remained identical to our previously published approach,^34^ including eye dissection, eye positioning within the clamp system, speckle pattern application, preconditioning, and imaging at the pressure steps of 5, 15, 30, and 45 mm Hg. With decreased image acquisition time, the automated S-DIC method allowed the user interaction with the system to be completely hands-off once the eye was properly installed. The S-DIC postprocessing techniques were also improved with the automated system through acquiring consistent angle sequence images between ocular experiments. This allowed for robust postprocessing scripts, less procedural variations between eyes, and faster generation of ocular strain fields.

To generate strain maps, strains were calculated following the protocol described in our previous study.^37^ Briefly, a geometry mesh was generated using measured position data triangulation. Larger elements were remeshed using custom Matlab code so that the element sizes for all regions and zones were similar. The Green–Lagrange strain tensor, E, was computed for each element using the equation \newcommand{\bialpha}{\boldsymbol{\alpha}}\newcommand{\bibeta}{\boldsymbol{\beta}}\newcommand{\bigamma}{\boldsymbol{\gamma}}\newcommand{\bidelta}{\boldsymbol{\delta}}\newcommand{\bivarepsilon}{\boldsymbol{\varepsilon}}\newcommand{\bizeta}{\boldsymbol{\zeta}}\newcommand{\bieta}{\boldsymbol{\eta}}\newcommand{\bitheta}{\boldsymbol{\theta}}\newcommand{\biiota}{\boldsymbol{\iota}}\newcommand{\bikappa}{\boldsymbol{\kappa}}\newcommand{\bilambda}{\boldsymbol{\lambda}}\newcommand{\bimu}{\boldsymbol{\mu}}\newcommand{\binu}{\boldsymbol{\nu}}\newcommand{\bixi}{\boldsymbol{\xi}}\newcommand{\biomicron}{\boldsymbol{\micron}}\newcommand{\bipi}{\boldsymbol{\pi}}\newcommand{\birho}{\boldsymbol{\rho}}\newcommand{\bisigma}{\boldsymbol{\sigma}}\newcommand{\bitau}{\boldsymbol{\tau}}\newcommand{\biupsilon}{\boldsymbol{\upsilon}}\newcommand{\biphi}{\boldsymbol{\phi}}\newcommand{\bichi}{\boldsymbol{\chi}}\newcommand{\bipsi}{\boldsymbol{\psi}}\newcommand{\biomega}{\boldsymbol{\omega}}\(E = {1 \over 2}({F^T}F - I)\), where \newcommand{\bialpha}{\boldsymbol{\alpha}}\newcommand{\bibeta}{\boldsymbol{\beta}}\newcommand{\bigamma}{\boldsymbol{\gamma}}\newcommand{\bidelta}{\boldsymbol{\delta}}\newcommand{\bivarepsilon}{\boldsymbol{\varepsilon}}\newcommand{\bizeta}{\boldsymbol{\zeta}}\newcommand{\bieta}{\boldsymbol{\eta}}\newcommand{\bitheta}{\boldsymbol{\theta}}\newcommand{\biiota}{\boldsymbol{\iota}}\newcommand{\bikappa}{\boldsymbol{\kappa}}\newcommand{\bilambda}{\boldsymbol{\lambda}}\newcommand{\bimu}{\boldsymbol{\mu}}\newcommand{\binu}{\boldsymbol{\nu}}\newcommand{\bixi}{\boldsymbol{\xi}}\newcommand{\biomicron}{\boldsymbol{\micron}}\newcommand{\bipi}{\boldsymbol{\pi}}\newcommand{\birho}{\boldsymbol{\rho}}\newcommand{\bisigma}{\boldsymbol{\sigma}}\newcommand{\bitau}{\boldsymbol{\tau}}\newcommand{\biupsilon}{\boldsymbol{\upsilon}}\newcommand{\biphi}{\boldsymbol{\phi}}\newcommand{\bichi}{\boldsymbol{\chi}}\newcommand{\bipsi}{\boldsymbol{\psi}}\newcommand{\biomega}{\boldsymbol{\omega}}\(F\) is the deformation gradient tensor and \newcommand{\bialpha}{\boldsymbol{\alpha}}\newcommand{\bibeta}{\boldsymbol{\beta}}\newcommand{\bigamma}{\boldsymbol{\gamma}}\newcommand{\bidelta}{\boldsymbol{\delta}}\newcommand{\bivarepsilon}{\boldsymbol{\varepsilon}}\newcommand{\bizeta}{\boldsymbol{\zeta}}\newcommand{\bieta}{\boldsymbol{\eta}}\newcommand{\bitheta}{\boldsymbol{\theta}}\newcommand{\biiota}{\boldsymbol{\iota}}\newcommand{\bikappa}{\boldsymbol{\kappa}}\newcommand{\bilambda}{\boldsymbol{\lambda}}\newcommand{\bimu}{\boldsymbol{\mu}}\newcommand{\binu}{\boldsymbol{\nu}}\newcommand{\bixi}{\boldsymbol{\xi}}\newcommand{\biomicron}{\boldsymbol{\micron}}\newcommand{\bipi}{\boldsymbol{\pi}}\newcommand{\birho}{\boldsymbol{\rho}}\newcommand{\bisigma}{\boldsymbol{\sigma}}\newcommand{\bitau}{\boldsymbol{\tau}}\newcommand{\biupsilon}{\boldsymbol{\upsilon}}\newcommand{\biphi}{\boldsymbol{\phi}}\newcommand{\bichi}{\boldsymbol{\chi}}\newcommand{\bipsi}{\boldsymbol{\psi}}\newcommand{\biomega}{\boldsymbol{\omega}}\(I\) is the identity matrix. \newcommand{\bialpha}{\boldsymbol{\alpha}}\newcommand{\bibeta}{\boldsymbol{\beta}}\newcommand{\bigamma}{\boldsymbol{\gamma}}\newcommand{\bidelta}{\boldsymbol{\delta}}\newcommand{\bivarepsilon}{\boldsymbol{\varepsilon}}\newcommand{\bizeta}{\boldsymbol{\zeta}}\newcommand{\bieta}{\boldsymbol{\eta}}\newcommand{\bitheta}{\boldsymbol{\theta}}\newcommand{\biiota}{\boldsymbol{\iota}}\newcommand{\bikappa}{\boldsymbol{\kappa}}\newcommand{\bilambda}{\boldsymbol{\lambda}}\newcommand{\bimu}{\boldsymbol{\mu}}\newcommand{\binu}{\boldsymbol{\nu}}\newcommand{\bixi}{\boldsymbol{\xi}}\newcommand{\biomicron}{\boldsymbol{\micron}}\newcommand{\bipi}{\boldsymbol{\pi}}\newcommand{\birho}{\boldsymbol{\rho}}\newcommand{\bisigma}{\boldsymbol{\sigma}}\newcommand{\bitau}{\boldsymbol{\tau}}\newcommand{\biupsilon}{\boldsymbol{\upsilon}}\newcommand{\biphi}{\boldsymbol{\phi}}\newcommand{\bichi}{\boldsymbol{\chi}}\newcommand{\bipsi}{\boldsymbol{\psi}}\newcommand{\biomega}{\boldsymbol{\omega}}\(E\) was used to calculate two in-plane principal strains for each element, E1 and E2. The strain calculations assumed that the 5-mm Hg base geometry was the undeformed state for each inflation pressure. This resulted in three pairs of elemental principal strains: E1 and E2 from 5 to 15, 5 to 30, and 5 to 45 mm Hg (simply referred to as pressures 15, 30, and 45 mm Hg for the remainder of this article).

### Zonal and Regional Segmentation

**Figure 1:**
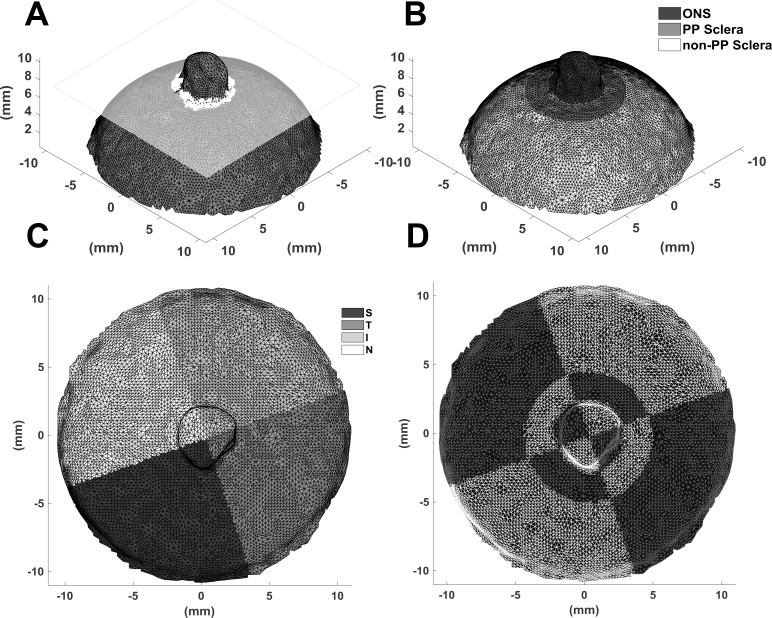
Posterior scleral pole showing (A) the dividing plane based on ONS saddle ring points and the division into (B) ONS, PP scleral, and non-PP scleral zones, (C) S, T, I, and N regions, and (D) 12 spatial locations defined by regions and zones.

To compare strain values from different spatial locations, all geometries were segmented into three zones and four regions. With regards to the zones, each geometry was divided into the ONS, the PP sclera, and the non-PP sclera. Briefly, the geometry was rotated so that the ONS pointed in the positive *z*-direction. A Hessian matrix was calculated along the geometry and was used to determine the mean curvature for each element. Using a curvature criterion, the saddle points of the ONS ring were determined and used to fit to a dividing plane. All elements above the plane were designated as ONS elements as shown in Figure 1A. The remaining elements within a distance of 2 mm below the plane were designated as PP scleral elements.^44^ The remainder of the scleral elements were designated as non-PP scleral elements, as shown in Figure 1B. After zonal segmentation, the ONS elements were imported into Rhinoceros 3D (Robert McNeel & Associates, Seattle, WA, USA), which was used to close the ONS geometry so that the volume of each ONS could be calculated for each sample (mm^3^). In addition, the diameter and length of the ONS were calculated. Due to the nonuniform shape of the ONS, the diameter was calculated for each sample by subtracting the maximum and minimum positions of the ONS in the *x*- and *y*-direction. Both of these measurements were averaged and recorded as the diameter (mm). Similarly, the maximum and minimum positions of the ONS were subtracted in the *z*-direction. This was recorded as the ONS length (mm). It should also be noted that the ONS zone for all eyes encompassed mostly the optic nerve sheath and a small portion of the optic nerve that was exposed during the enucleation process.

For regional definition, the geometry was divided into S, I, T, and N. To do this, the centroid of the scleral opening produced by the dividing plane was set as the origin of the geometry. Furthermore, the geometry was rotated so that the normal of the dividing plane was aligned with the *z*-axis. The nodes of the all elements were converted from Cartesian to spherical coordinates. The known S direction was set as 0° in the meridional direction. All elements within 45° of the S direction were designated as S elements. The same was done for the remaining regions dividing the whole geometry into four equally spaced regions as shown in Figure 1C. The orientation of the T and N direction was determined by whether the eye was oculus dextrus (OD) or oculus sinister (OS). The overall 12 spatial locations defined by regions and zones are shown in Figure 1D.

### Optic Nerve Axon Counting

**Figure 2:**
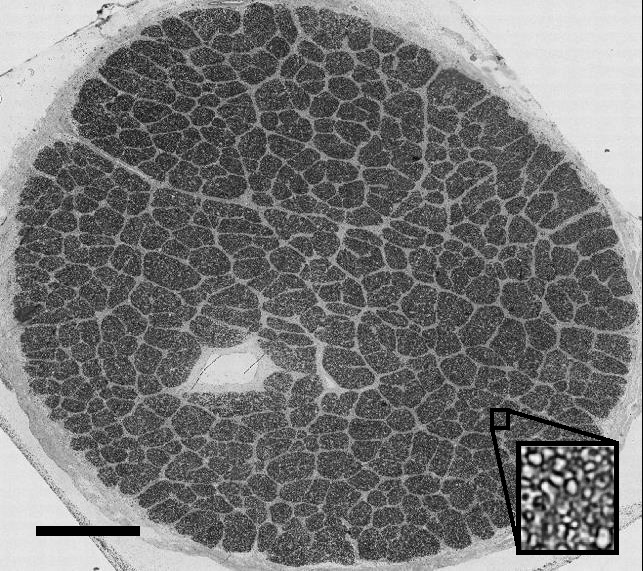
Microscope image of full optic nerve cross section used for semiautomated axon counting. Dark sections indicate bundles of axons separated by lighter connective tissue. Scale bar denotes 500 μm.

The embedded optic nerve cross sections were visualized on a Nikon Eclipse 90i microscope using a montaging method with Nikon NIS-Elements software (Nikon, Inc., Millville, NY, USA). The method used individually imaged 60× magnifications with autofocus capabilities and a 15% overlap. A complete montage of an axon cross section is shown in Figure 2. Semiautomated axon counts were executed using image processing techniques in MatLab and methodology adapted from literature.^45,46^ The user was first prompted to identify the bounds of the optic nerve cross section to crop the image. Contrast-limited adaptive histogram equalization was applied to the cropped image to improve user accuracy of axon identification. The cropped portion of the image was divided equally into a sectional grid comprised of 200 sections horizontally by 200 sections vertically. From these 40,000 sections, 10 individual sections of axons were randomly selected and presented one at a time, and the user was prompted to manually click on the axons in each section. These manual counts were averaged across all 10 counted sections, providing an average axon density for the small image size. This was done to create a more accurate representation of the average axon density in these sections with respect to the entire optic nerve area, as axon distribution is somewhat nonuniform by nature. This calculated density was then used to extrapolate across the entire cross section to give an estimated axon count for the whole nerve. Each image was “counted” through the program three times to improve repeatability and accurate averages with SDs of axon counts being calculated for all nerves. Multiple users performed axon counts on all samples, and their counts were averaged to account for interuser variability.

### Statistical Analysis

A statistical analysis was performed using scripts written in R (GNU General Public License, R Core Team, Vienna, Austria). Specifically, a parsimonious linear mixed model built to account for repeated measures (lmer) was used. Racioethnicity (AD, ED, and HIS), zone (ONS, PP sclera, and non-PP sclera), region (S, T, I, and N), and pressure (15, 30, and 45 mm Hg) were considered fixed repeated-measure variables. For this model, pressure was considered as a discrete variable to evaluate racial and zonal differences at specific inflation pressures. Each donor was considered an individual subject for the repeated measures portion of the model. Mean E1 and E2 estimates were determined for each spatial location at every inflation pressure. The data were arranged in a factorial form suitable for the statistical model. All data were normalized using a two-step SPSS Statistics (IBM Corporation, Armonk, NY, USA) transformation procedure that used inverse distribution functions.^47^ Post hoc mean E1 and E2 estimates were calculated using an R least squares mean function (lsmeansLT). Pairwise comparisons were calculated using an R differences of least squares means function (difflsmeans). Both of these functions used the R linear mixed model previously mentioned. To account for familywise error, a Bonferroni-type correction was applied on any pairwise test conducted.

For regression analysis, pressure was considered as a continuous variable to determine the relationship of E1 and E2 as a function of pressure for every zone for each racioethnic group. This was done using the linear mixed model in SPSS. Similar to the R model already mentioned, zone and region were considered repeated-measure variables, and each donor considered an individual subject for the model. Additionally, the same regression model was used to determine the relationships of mean E1 and mean E2 at all spatial locations and inflation pressures as a function of ONS volume and optic nerve axon count.

## Results

### E1 and E2 Strain Maps

**Figure 3:**
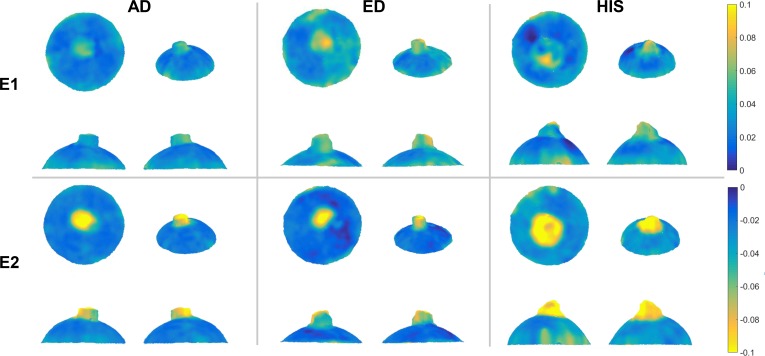
A representative example of E1 and E2 strain maps at 45 mm Hg from each racioethnic group. Four views are included for each sample: top view (upper left), isoparametric view (upper right), back side view (lower left), and front side view (lower right). Note: the color map for the E2 strain maps has been reversed compared with the E1 strain maps to emphasize absolute value.

The E1 and E2 magnitudes for the 5- to 45-mm Hg inflation of one representative sample from each racioethnic group are shown in Figure 3. The E1 and E2 magnitudes in the ONS region were qualitatively larger than that of the scleral zones for all racioethnic groups. E2 magnitudes (compressive) were qualitatively higher than that of E1 magnitudes (tensile) in the same region. Furthermore, more qualitative regional heterogeneity can be observed in the ED and HIS samples compared with the AD sample. Overall, the reported values of E1 and E2 in the scleral zones were consistent with values reported in the literature.

### Statistical Analysis

#### Pressure as Discrete Variable

**Figure 4:**
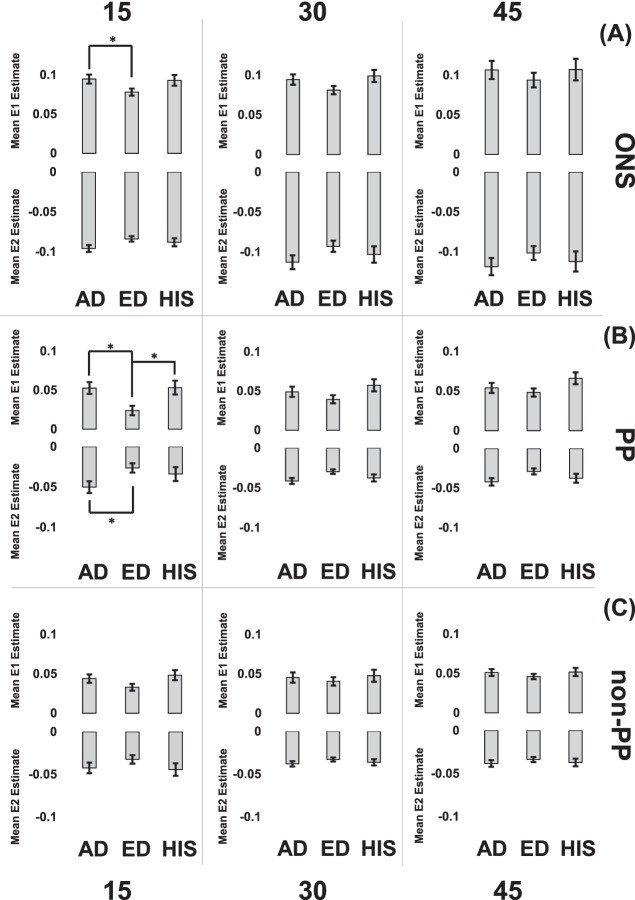
Mean E1 and E2 value estimate zonal comparisons for all inflation pressures for each racioethnic group. *P < 0.05. Error bars denote SE as estimated by the statistical model.

A linear mixed model was performed in R using pressure as a discrete variable. The R linear mixed model results showed that all interaction terms higher than a second-order interaction were not significant and were therefore removed from the main model expression. The mean E1 and E2 estimates, which represent the values predicted by the statistical model, were calculated, and pairwise comparisons were performed between racioethnic groups for all three zones at each inflation pressure. The zonal comparisons are shown in Figure 4.

In the ONS (Fig. 4A), the mean E1 values for AD eyes were significantly higher than that of ED eyes (*P* = 0.024) at 15 mm Hg. Similarly, the mean E2 absolute values were significantly higher for AD eyes compared with that of ED at 15 mm Hg. No significant zonal differences across racioethnic groups were shown at 30 and 45 mm Hg for both mean E1 and E2 values. For the PP scleral zone (Fig. 4B), the mean E1 values for ED eyes were significantly lower than that of AD and HIS eyes (*P* = 0.0219 and 0.0387, respectively) at 15 mm Hg. No significant racioethnic differences were observed at 30 and 45 mm Hg in the PP scleral zone. The mean E2 values were significantly higher for AD eyes compared with ED eyes at 15 mm Hg (*P* = 0.049). For the non-PP scleral zone (Fig. 4C), there were no significant racioethnic differences for mean E1 or mean E2 values at all inflation pressures.

There were few significant regional differences within racioethnic groups at different inflation pressures. In the PP zone for HIS eyes, the mean E2 absolute values for the T region was lower than that of the N region at 15 mm Hg (*P* = 0.048, respectively). For the non-PP scleral zone, the ED mean E1 values for I region were higher compared with the S region for 30 mm Hg (*P* = 0.048). For the mean E2 absolute values, the I region was significantly higher than the T region at 30 and 45 mm Hg for ED eyes (*P* = 0.024 and 0.03, respectively). All other mean E1 and mean E2 regional differences within racioethnic groups were not significant. For zonal differences within racioethnic groups, the mean E1 values and mean E2 absolute values for the ONS zone were significantly higher than that of both the PP and non-PP scleral zone for all racioethnic groups at all inflation pressure (*P* < 0.001 for all). All other mean E1 and mean E2 differences between PP and non-PP scleral regions within racioethnic groups were not significant.

Pairwise comparisons were made between different pressure states for each zone within each racioethnic groups. For the AD eyes in the ONS zone, E1 values at 30 mm Hg were significantly lower than that of 45 mm Hg (*P* = 0.033) and the E2 absolute values at 15 mm Hg were significantly lower than that of 45 mm Hg (*P* = 0.012). In the non-PP zone, the E1 values for AD eyes at 15 mm Hg were significantly lower than that of 45 mm Hg (*P* < 0.001). For ED eyes, the ONS mean E1 values at 45 mm Hg were significantly higher than that of 30 and 15 mm Hg (*P* = 0.045 and <0.001, respectively). The ONS mean E2 absolute values at 15 mm Hg were significantly lower than that of 45 mm Hg (*P* = 0.026). In the PP scleral zone, the ED mean E1 values at 15 mm Hg was significantly lower than that of 30 and 45 mm Hg (*P* = 0.033 and <0.001, respectively). Furthermore, the mean E1 values for ED eyes at 30 mm Hg in the PP scleral zone was significantly lower than that of 45 mm Hg (*P* = 0.015). As for the non-PP scleral zone, the mean E1 values for ED eyes at 15 mm Hg were significantly lower than that at 45 mm Hg (*P* = 0.026). As for HIS eyes, in the ONS zone, the mean E2 absolute values at 15 mm Hg were significantly lower than that at 45 mm Hg (*P* = 0.023). In the PP scleral zone, the mean E1 values at 15 mm Hg were significantly lower than that at 45 mm Hg (*P* = 0.002). In the non-PP scleral zone, the mean E1 values at 15 mm Hg were significantly lower than that at 45 mm Hg (*P* = 0.028). All other comparisons in all zones for all racioethnic groups were not significantly different.

#### Pressure as Continuous Variable

**Figure 5:**
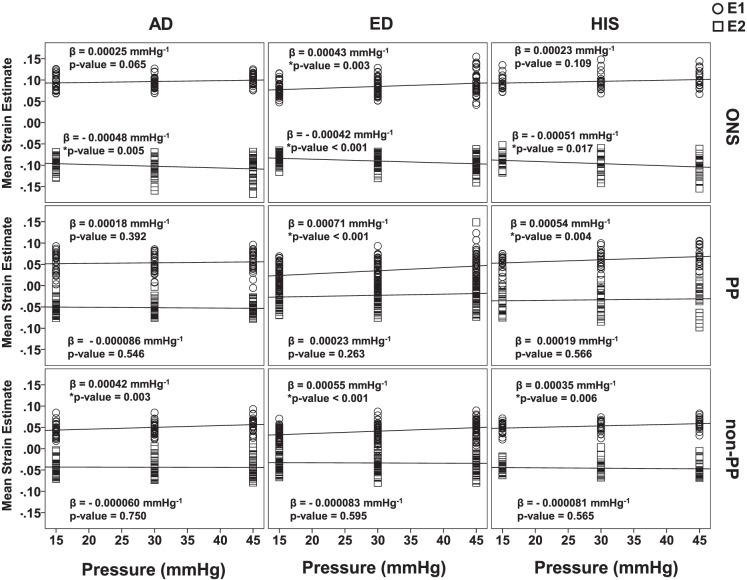
Regression plots of mean E1 and E2 values as a function of pressure for all racioethnic groups within each zone.

A mixed linear model was performed in SPSS using pressure as a continuous variable to determine the relationship of mean E1 and E2 values as a function of pressure. This was done for each zone for each racioethnic group. The regression results are shown in Figure 5. In the ONS, only ED eyes had a significant positive relationship between mean E1 values and pressure (*P* = 0.003). AD, ED, and HIS eyes had a significant positive relationship between mean E2 absolute values and pressure (*P* = 0.005, *P* < 0.001, and *P* = 0.017, respectively). In the PP scleral zone, both ED and HIS eyes had a positive relationship between mean E1 values and pressure. Furthermore, the slope of the mean E1 pressure relationship of the ED eyes was significantly higher than that of AD eyes (*P* = 0.04). For mean E2 values, there was no significant relationship with pressure for any of the racioethnic groups. As for the non-PP scleral zone, AD, ED, and HIS eyes had a significant positive relationship between mean E1 values and pressure (*P* = 0.003, *P* < 0.001, and *P* = 0.006, respectively).

#### Axon Count

**Figure 6:**
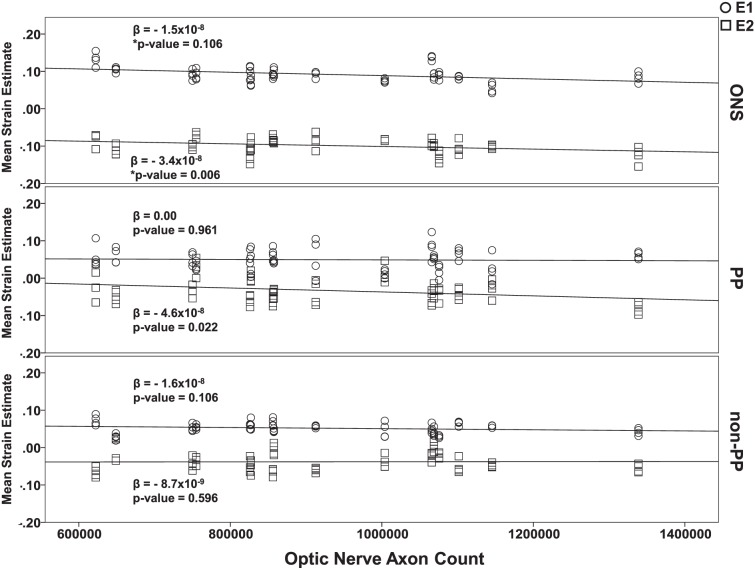
Regression plots of mean E1 and E2 values as a function of optic nerve axon count at 45 mm Hg within each zone.

The mean axon counts were 9.05 × 10^5^ ± 1.2 × 10^5^, 9.65 × 10^5^ ± 2.1 × 10^5^, 1.1 × 10^6^ ± 3.0 × 10^5^ for the AD (*n* = 4), ED (*n* = 10), and HIS (*n* = 2) donors, respectively, where the ranges indicate SDs. A 2-tailed Student's *t*-test was performed comparing the AD and ED eyes, which showed that there was no significant difference between both groups in regard to the optic nerve axon count (*P* = 0.964). A mixed linear model was performed in SPSS using axon count as a continuous variable to determine the relationship of mean E1 and E2 values for all eyes as a function of axon count for all three zones at 15, 30, and 45 mm Hg. In the ONS zone, there was a significant negative relationship between mean E1 values and axon count at 45 mm Hg (*P* = 0.002). There was a significant positive relationship between mean E2 absolute values and axon count at 45 mm Hg (*P* = 0.006). In the PP scleral zone at 45 mm Hg, there was no significant relationship between mean E1 values and pressure (*P* = 0.961); however, there was a significant positive relationship between E2 absolute values and axon count (*P* = 0.022). In the non-PP scleral zone at 45 mm Hg, there were no significant relationships of neither mean E1 values nor mean E2 values with axon count (*P* = 0.106 and 0.596, respectively). All relationships between mean E1 and E2 and axon counts at 15 and 30 mm Hg at all zones were shown to be not significant. The regression plots for all zones at 45 mm Hg are shown in Figure 6.

#### ONS Dimensions

**Figure 7:**
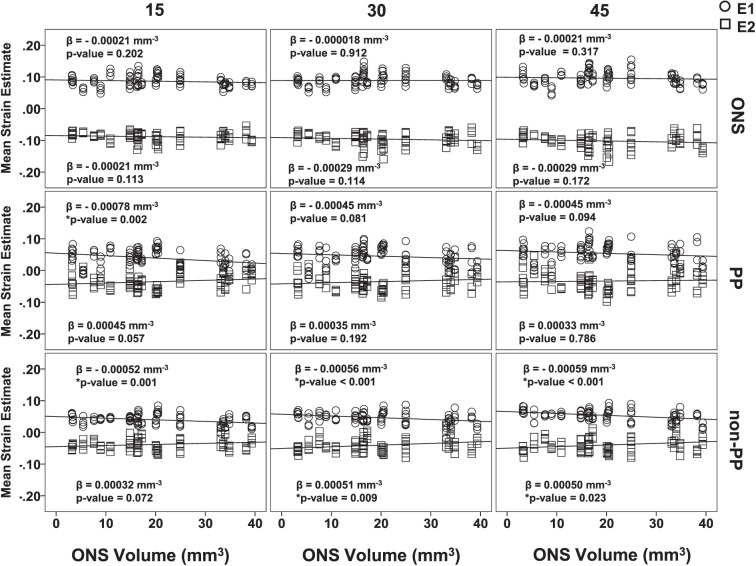
ONS volume (left), diameter (middle), and length (right) comparisons between racioethnic groups. Error bars denote 95% confidence interval.

The mean ONS volumes were 16.2 ± 6.0, 23.6 ± 7.9, and 17.1 ± 11.9 mm^3^ for the AD, ED, and HIS donors, respectively. The mean ONS diameters were 5.2 ± 0.4, 6.4 ± 0.7, and 6.3 ± 1.7 mm for the AD, ED, and HIS donors, respectively. The mean ONS lengths were 2.9 ± 0.4, 3.2 ± 0.7, and 6.3 ± 0.9 mm for the AD, ED, and HIS donors, respectively. All ranges for all dimensions indicate 95% confidence intervals. Single-factor ANOVAs concluded that there were no significant differences between the different racioethnic groups in regard to the ONS volume (*P* = 0.330), diameter (*P* = 0.154), and length (*P* = 0.632). Comparisons of ONS volume, diameter, and length among racioethnic groups are shown in Figure 7.

**Figure 8:**
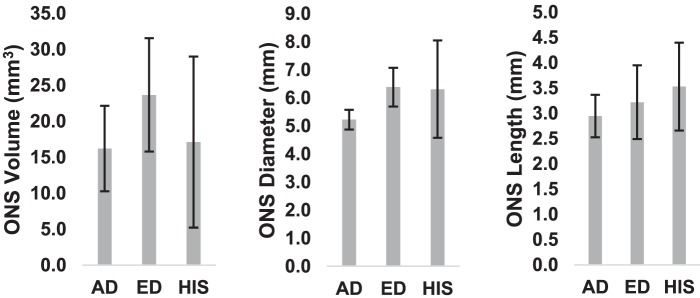
Regression plots of mean E1 and E2 values as a function of ONS volume for all inflation pressures within each zone.

In addition, to evaluate the effect of the ONS size on the E1 and E2 values, a mixed linear model was performed in SPSS determine the relationship of mean E1 and E2 values for all eyes as a function of ONS volume for all three zones at 15, 30, and 45 mm Hg. The results are shown in Figure 8. Overall, it was found that the mean E1 and mean E2 value did not have a significant relationship with the ONS volume in the ONS zone. As for the PP scleral zone, the mean E1 values had a significant negative relationship with the ONS volume at 15 mm Hg only (*P* = 0.002). As for the non-PP scleral zone, there was a significant relationship between mean E1 and ONS volume at all pressures (*P* < 0.001 for all). Furthermore, there was also a significant negative relationship between mean E2 absolute values and ONS volume at 30 and 45 mm Hg (*P* = 0.009 and 0.023, respectively) in the non-PP scleral zone.

## Discussion

A zonal racioethnic in-plane principal strain comparison was conducted across three inflation pressures for normal human scleral shells using displacement measurements collected via S-DIC. The ONS tensile and compressive strains for the ED eyes were significantly lower than that of AD eyes at 15 mm Hg (Fig. 4A). The PP scleral compressive strains for the AD eyes were significantly larger than that of ED eyes at 15 mm Hg (Fig. 4B). For ED and HIS eyes, tensile strains in the ONS and non-PP scleral zones increased significantly with pressure, whereas AD eyes showed a significant relationship with pressure in the non-PP scleral zone only (Fig. 5). Additionally, only ED and HIS eyes had a significant positive relationship between tensile strain in the PP scleral region, whereas AD PP scleral strains did not have a significant relationship with pressure. All racioethnic groups had significant compressive strain relationships with pressure in the ONS zone (Fig. 5).

Several studies have previously used principal strains as an end point to compare spatial locations in eyes,^13,29,48,49^ whereas some used circumferential and meridional strain.^21,50,51^ The principal strain values reported in our study were found to be within the range of principal strain values shown in other studies. Fazio et al.^29^ used ESPI to investigate the mean maximum principal strain (E1) regional variability in the PP and midperipheral scleral regions (each divided into eight meridional regions) in 10 pairs of normal eyes ages 57 to 90 years old with nonspecified racioethnic classification. The study by Fazio et al.^29^ showed that the I region demonstrated E1 strain values higher than the S and N regions, which was consistent with our regional trend of the ED tensile strains in the non-PP scleral zone. Coudrillier et al.^51^ found regional differences in fiber alignment in the PP scleral location for normal eyes (*n* = 9). In addition, our research group has previously investigated regional microstructural differences for glaucomatous and normal human eyes of ED.^52^ In that study, Danford et al.^52^ showed that normal ED eyes exhibited lower percent of equatorial fiber organization in the I region compared to all other regions, especially as compared with the N region, which had the highest degree of circumferential fiber alignment. This disparity may explain the higher tensile and compressive strains in the I region of ED samples reported in the current study as a larger degree of noncircumferential fibers may lead to increased fiber realignment into the circumferential direction and subsequently larger in-plane compressive strains. Future microstructural information in AD and HIS eyes could further explain the various regional strain differences observed throughout all racioethnic groups. These studies are currently ongoing in our laboratory.

With regard to zonal differences within racioethnic groups, the study by Fazio et al.^29^ found that the PP sclera exhibited significantly higher mean maximum values of principal strains (E1) than midperipheral scleral regions for a 5- to 45-mm Hg pressure inflation. This is not consistent with our results that showed tensile differences only between the ONS and the both scleral zones at all pressures. The study by Fazio et al.^17^ also reported that PP sclera exhibited significantly higher values of mean E1 strains than midperipheral sclera for both donors of AD and ED. This was also in contrast to our results that indicated that the PP sclera did not differ significantly from the non-PP region for AD and ED eyes. This could be due to our study including non-PP sclera beyond the midperipheral scleral zone. Furthermore, the ONS volume of eyes in our study seemed to have a significant effect on the tensile and compressive strain values in the non-PP scleral zone (Fig. 8). This could further explain why our ED and AD zonal comparison results differed from those of Fazio et al.^17^ who did not include the ONS. In addition, the discrepancy between our research groups in zonal differences within racioethnic groups could be explained by a number of other factors, including scleral thickness variations. For example, a study by Norman et al.^49^ showed that strain differences between PP and non-PP strains could be attributed to the thickness increases adjacent to the optic nerve head. This was also confirmed by Coudrillier et al., who measured scleral thickness and reported a different stiffness between the PP and mid-posterior scleral regions for both normal and glaucomatous eyes,^21^ which they attributed to the PP sclera having lower degree of fiber alignment and lower mechanical anisotropy compared with midposterior scleral regions.^50^

To the author's knowledge, Fazio et al.^17^ and Grytz et al.^18^ are two of the few research groups that have studied racioethnicity as a factor in normal posterior scleral material properties using ESPI. In a study by Grytz et al.,^18^ the authors report that the in-plane strain, which they describe as the strain tangent to the scleral shell surface, was found to be significantly lower in donors of AD compared with ED in the PP scleral region (*P* = 0.015) for age groups of 20 to 90 and 23 to 73 years old, respectively, inflated from 5 to 45 mm Hg. The scleral strain results from Grytz et al.^18^ were not consistent with our results, which showed significantly lower PP scleral tensile strain for ED samples compared with AD samples at 15 mm Hg (*P* = 0.024). Furthermore, the PP scleral compressive strains for the ED samples were significantly smaller than that of AD at 15 mm Hg (*P* = 0.049; Fig. 4B). Discrepancies between our results and those of Grytz et al.^18^ could be attributed to differences in scleral thickness. Grytz et al.^18^ found no significant difference in scleral thickness between ED and AD eyes. However, racioethnic differences in scleral thickness could have contributed to the discrepancies that we observe between our different groups. Our laboratory is currently analyzing x-ray microcomputed tomography (μCT) images of the same scleral shells used in this study. Our preliminary results suggest that the sclera of ED eyes may be thicker than that of AD eyes, which would explain why the PP scleral strains of ED eyes would be smaller than that of AD eyes in our study. Grytz et al.^18^ used ultrasound to obtain thickness at 20 points for each eye (*n* = 40 for ED 20 to 90 years old, *n* = 22 for AD 23 to 73 years old). Our eyes are restricted to donors older than 50 years old, and the sample number in our study is lower than that of Grytz et al.^18^ The differences in thickness measurements between the racioethnic groups could be due to differences in measurement technique, age, the source, and/or inherent variability. Furthermore, the eyes used by Grytz et al.^18^ had their optic nerves severed flush, which as was previously suggested could significantly alter scleral deformation. Hence, comparing these two results may not be appropriate without accounting for these main differences in method and eye geometry. Grytz et al.^18^ used thickness data in FE simulations to calculate the shear moduli and estimate stiffness. AD eyes were shown to have a higher shear modulus than those of ED.^18^ The study by Fazio et al.^17^ found that age-related stiffening was significantly greater in the PP sclera for donors of AD compared with donors of ED. Both these studies are consistent with our results where the slope of the tensile strain–pressure relationship of the ED eyes was significant, whereas that of AD eyes was not, suggesting that AD eyes deformed less with pressure (stiffer) compared with ED eyes.

All axon counts were within ranges that have been considered normal in different studies in the literature.^53–60^ Both axon count and ONS volume were found to have significant effects on tensile and/or compressive strains in the different zones for at least one level of pressure. One possible concern may be that the biomechanical differences found between racioethnic groups could be due to racioethnic differences in ONS volume in our enucleated eyes. However, a statistical comparison on ONS volume (Fig. 7) showed no significant differences between racioethnic groups that would explain differences in tensile and compressive strains. Additionally, it is noteworthy that a significant correlation existed between axon count and strains at 45 mm Hg for normal nonglaucomatous eyes (Fig. 6). The fact that this correlation was found in normal eyes suggests that axon loss may be a normal ongoing process in humans. It remains to be seen if this relationship plays a role in the predisposition of a person to glaucomatous damage. Furthermore, it seems that as proximity to the ONS decreases, the effect of axon count on strain diminishes (from ONS to PP to non-PP). It also seems that a loss of axons is associated with increased deformation in the optic nerve, especially at higher pressures. Further research will be needed to ascertain whether axon loss itself plays a role in exacerbating the biomechanical insult in primary open angle glaucoma.

There are several limitations to the work presented here. Although our study does provide ONS and PP scleral strain information, the reported strain is only surface strain and does not provide information regarding the mechanical environment at the LC, which has been shown to be an important region as it relates to RGC damage. On that note, the authors also wish to recommend that interpretations of the ONS strain results reported here not be construed as “optic nerve deformations” but rather as strains of mostly the sheath surrounding the optic nerve and a small portion of the optic nerve that was exposed during enucleation. It should be noted that all optic nerve stumps most likely were swollen in our experiments compared with in vivo geometry. However, the well-controlled temperature and humidity environment and consistent preconditioning at all inflation pressures did not allow for any additional swelling during SDIC imaging. Unavoidable issues related to tissue edema and a somewhat nonphysiologic unconstrained severed nerve stump should serve as caution to the reader when interpreting these results. That being said, the fact that the presence (and volume) of the ONS volume did influence peripapillary scleral deformation indicate its inclusion may be important in future studies. Our study also focused entirely on assessing normal eyes and did not include glaucomatous eyes of any race or ethnicity. Future studies should be designed to study more thoroughly how glaucoma differentially affects posterior pole deformations in these populations, especially in regions close to the ONS. Furthermore, due to errors in nerve processing, our research group was unable to measure the axon counts for all the eyes used in S-DIC in this study. Therefore, the axon count relationship with strains is restricted to a subset of the eyes analyzed for strain. Last but not least, the authors wish to convey the limited sample sizes used in this study and recommend the reader interpret the presented results and conclusions with this in mind.

Although there is clinical evidence that both people of AD and HIS are more predisposed to developing glaucoma compared with people of ED, ocular biomechanical studies of AD and particularly HIS scleral shells are not common in the literature. Our results showed that those of HIS exhibited PP scleral tensile strains that were significantly different than those of ED at 15 mm Hg. HIS tensile strains in the ONS region did not have a significant relationship with pressure, whereas ED eyes did. However, the behavior of tensile and compressive strains in both PP and non-PP scleral zones did not differ significantly between ED and HIS eyes. Preliminary results from our ongoing research (data not shown) suggest that ED eyes may also have significantly thicker sclera that of HIS eyes. The lack of consistency between HIS and AD strain patterns compared with ED suggests that the mechanism of higher prevalence of OAG in AD eyes could be different than that of HIS. More comprehensive studies are necessary to investigate possible alternative explanations to prevalence of ocular disease in both people of AD and HIS. In addition, it is important to note that these results show increasing pressure above 15 mm Hg did not result in additional strain in the PP for AD eyes. This may be due to the nonlinear stress–strain material behavior presenting a more prominent strain-stiffening effect in that zone for AD eyes, which is considered a higher-risk group. In future work, it would be interesting to explore whether glaucomatous eyes demonstrate this type of behavior and how this may play a role in the incidence or progression of glaucoma. Another prominent result of our study was that the ONS strains were much higher than the scleral strains at every IOP for all racioethnic groups (Fig. 4). Although it should be noted that the strains measured on the ONS in this study should not be interpreted as identical to those occurring in vivo, this result strongly confirms that there is an IOP-dependent mechanical link between the peripapillary sclera and the dural sheath. This link may include bending modes of the sclera/dural sheath and may contribute to the overall biomechanical environment of the optic nerve head. Future research will need to be performed to more directly test the influence of the dural sheath on the optic nerve head mechanical response in OAG.

Our study is the first determine strains for all zones and regions of donors of HIS. Overall, we found that the HIS PP scleral tensile strain relationship with pressure were more similar to those of ED than AD for tensile strains. Our study is also the first to map the strain behavior of the human ONS for any racioethnic group using S-DIC. We were also the first to find a significant relationship between tensile and compressive strains and ONS volume and axon count. This shows that studying the strains of ONS, which serves as the sheath for nerves, may be an important consideration in biomechanical evaluation of the posterior pole. The results in this study indicate that keeping ONSs attached to their scleral shells, while accounting for volume and axon count, may be necessary to conduct a more complete ocular biomechanical analysis. Future studies by our group will combine geometry data collected via μCT with strain data collected from this study to develop an inverse finite element model, which will investigate if the strain differences quantified in this study correspond to true mechanical property differences.
